# Dental Association

**Published:** 1864-05

**Authors:** S. B. Palmer

**Affiliations:** Rec. Secretary. Tully


### DENTAL ASSOCIATION.

The Annual Meeting of the "Central New York Dental Association will be held in Auburn, on Tuesday, May 10th. 1864, commencing at 2 o'clock, P. M., and continuing two days,

ORDER OF BUSINESS.

1st. Preservation of natural teeth:—To what extent is it practicable? How best accomplished?

2d. Alveolar Abscess.

3d. Treatment of exposed Nerves.

4th. Mechanical Dentistry.

5th. Miscellaneous Business.

The reading of written arguments upon any of the subjects under discussion will be in order.

All Practicing Dentists in Central New York are invited to join the Association, and take part in the exercises.

S. B. PALMER, *Rec. Secretary.*

*Tully, April* 11, 1864.

